# The Effectiveness of High-frequency Repetitive Transcranial Magnetic Stimulation in Persistent Somatic symptoms Disorder: A Case report study

**DOI:** 10.1192/j.eurpsy.2024.1342

**Published:** 2024-08-27

**Authors:** M. K. Albalushi

**Affiliations:** Psychiatry, Oman medical specialty board, Muscat, Oman

## Abstract

**Introduction:**

*Background*:

Somatic symptoms disorders are usually comorbid with depressive disorders despite that there is little evidence for effective treatment for it. Repetitive transcranial magnetic stimulation (rTMS) have been approved by FDA for mildly resistance depression. From this point we hypothesized that rTMS delivered over the prefrontal cortex (PFC) may be useful in somatic symptoms disorder. Therefore, in our case report we want to shed light on the potential effectiveness of rTMS in somatic symptoms disorder.

**Objectives:**

case report

**Methods:**

case report

**Results:**

*Case Report*:

A 65-year-old Omani female with multiple medical comorbidities on multiple medications. She presented complaining of multiple somatic complains in the last 2 years after visiting multiple clinics and underwent several specialists’ examinations, investigations and procedure for somatic treatments, all of them where normal.

Then patient was seen by different psychiatric clinic multiple anti-depressant and adjuvant anti-psychotic medication were try, patient still not improve.

Patient get admitted to hospital for observation and management. Initially she was preoccupying by her somatic complain kept on Fluoxetine and Olanzapine along with that topiramate was added, but still with minimal improvement. Then rTMS was added to her management plan following Intermittent theta burst (iTBS) rTMS protocol. After complete all sessions of rTMS patient was recovering from her all symptoms, no complain report from her.

**Conclusions:**

*Conclusion*: our case highlights the important of investigated more thoroughly in rTMS as treatment option for Persistent Somatic symptoms Disorder.

**Disclosure of Interest:**

None Declared

